# Ivermectin for the prevention of COVID-19: addressing potential bias and medical fraud

**DOI:** 10.1093/jac/dkac052

**Published:** 2022-02-22

**Authors:** Andrew Hill, Manya Mirchandani, Leah Ellis, Victoria Pilkington

**Affiliations:** 1 Department of Pharmacology and Therapeutics, University of Liverpool, Liverpool L7 3NY, UK; 2 Faculty of Medicine, Imperial College London, London, UK; 3 Oxford University Clinical Academic Graduate School, University of Oxford, Oxford, UK

## Abstract

**Background:**

Ivermectin is an antiparasitic drug being investigated in clinical trials for the prevention of COVID-19. However, there are concerns about the quality of some of these trials.

**Objectives:**

To conduct a meta-analysis with randomized controlled trials of ivermectin for the prevention of COVID-19, while controlling for the quality of data. The primary outcome was RT–PCR-confirmed COVID-19 infection. The secondary outcome was rate of symptomatic COVID-19 infection.

**Methods:**

We conducted a subgroup analysis based on the quality of randomized controlled trials evaluating ivermectin for the prevention of COVID-19. Quality was assessed using the Cochrane risk of bias measures (RoB 2) and additional checks on raw data, where possible.

**Results:**

Four studies were included in the meta-analysis. One was rated as being potentially fraudulent, two as having a high risk of bias and one as having some concerns for bias. Ivermectin did not have a significant effect on preventing RT–PCR-confirmed COVID-19 infection. Ivermectin had a significant effect on preventing symptomatic COVID-19 infection in one trial with some concerns of bias, but this result was based on *post hoc* analysis of a multi-arm study.

**Conclusions:**

In this meta-analysis, the use of ivermectin was not associated with the prevention of RT–PCR-confirmed or symptomatic COVID-19. The currently available randomized trials evaluating ivermectin for the prevention of COVID-19 are insufficient and of poor quality.

## Introduction

COVID-19 was declared a pandemic by the WHO in March 2020.^1^ In the early stages of the pandemic, there were multiple uncertainties regarding the timeline of vaccine development and production.^2^ Therefore, investigational drugs were being assessed for the prevention of COVID-19, for example the REGEN-COV monoclonal antibody therapy, which was granted emergency use authorization as post-exposure prophylaxis by the FDA in October 2021.^3^

Although vaccines have been approved for use since January 2021, several challenges face worldwide uptake.^4^ Additionally, individuals who are immunocompromised are contraindicated for the COVID-19 vaccine.^5^ Furthermore, transmission of SARS-CoV-2 can continue to occur despite an individual being vaccinated.^6^ This suggests that a drug could play an additional role in the prevention of COVID-19.

Clinical trials are being conducted globally to evaluate the efficacy and safety of drugs to prevent COVID-19 infection. For example, molnupiravir is currently being evaluated in the MOVe-AHEAD trial.^7^ However, there are concerns regarding the quality of some of these trials evaluating drugs for COVID-19. For example, an observational study on hydroxychloroquine for COVID-19, published in *The Lancet*, was retracted due to concerns about the validity of the data.^8^ The company providing the data for this study, Surgisphere, claimed to have access to patient data. However, on further investigation, major inconsistencies were identified in the data, suggesting it was fabricated.^8^

Ivermectin is an FDA-approved antiparasitic drug, which was shown to have antiviral effects against SARS-CoV-2 *in vitro*.^9^ Following this, ivermectin has been evaluated for re-purposing against SARS-CoV-2 in clinical trials globally. Our earlier analysis suggested that the significant effect of ivermectin on the treatment of COVID-19 was based on high-risk and potentially fraudulent studies.^10^ Therefore, the purpose of this review was to analyse randomized controlled trials (RCTs) of ivermectin for the prevention of COVID-19, while controlling for the quality of data.

## Methods

The systematic review and meta-analysis were conducted according to PRISMA guidelines. Systematic searches were conducted on Clinicaltrials.gov, PubMed, Embase, MedRxiv, Research Square and the WHO International Clinical Trials Registry Platform to identify RCTs evaluating ivermectin for the prevention of SARS-CoV-2 infection. In addition to the standard Cochrane risk of bias tool (RoB 2), a detailed assessment of study quality was performed.^11^ Firstly, we evaluated trials based on the effectiveness of their randomization process by comparing baseline characteristics across treatment arms using the chi-squared test. Secondly, randomization dates were checked to ensure patients were randomized into the treatment arms on similar dates. Thirdly, checks were conducted to evaluate whether recruitment to treatment arms was balanced at each investigational centre. Furthermore, we analysed patient-level databases, where available, to check for any evidence of duplicate participants or unexpected homogeneity or heterogeneity. From this, a meta-analysis was conducted with subgroups of clinical trials at different risk-of-bias levels. The individual trial statistics were pooled using the random-effects inverse-variance model. The significance threshold was set at 5% (two-sided) and all analyses were conducted through RevMan 5.3. The primary outcome was RT–PCR-confirmed COVID-19 infection. The secondary outcome was the rate of symptomatic COVID-19 infection.

### Ethics

All the clinical trials included in the meta-analysis were approved by local ethics committees and all patients gave informed consent.

### Quality assessment

Four studies met the criteria and were included in the meta-analysis (Table 1). Two studies were conducted in Egypt,^12,13^ one in Argentina^14^ and one in Singapore.^15^ The studies included a range of participants, for example high-risk migrant workers living in dormitories, healthcare personnel and close contacts. The duration of treatment with ivermectin ranged between 1 day and once per week for 4 weeks across the studies. The meta-analysis included a total of 1974 participants.

**Table 1.** Studies included in the meta-analysis

Study	Risk of bias	Treatment arm	RT–PCR-confirmed infection *n*/*N* (%)	Symptomatic infection *n*/*N* (%)
Elgazzar^12^ (Egypt)	Potential fraud	Ivermectin	2/100 (2)	—
		Control	10/100 (10)	—
Shoumann^13^ (Egypt)	High risk	Ivermectin	—	15/203 (7.4)
		Control	—	59/101 (58.4)
Chahla^14^ (Argentina)	High risk	Ivermectin	4/117 (3.4)	—
		Control	25/117 (21.4)	—
Seet^15^ (Singapore)	Some concerns	Ivermectin	90/617 (14.6)	32/398 (8)
		Hydroxychloroquine	32/432 (7.4)	29/212 (13.7)
		Povidone-iodine	50/735 (6.8)	42/338 (12.4)
		Zinc plus vitamin C	50/634 (7.9)	33/300 (11)
		Vitamin C	85/619 (13.7)	64/433 (14.8)

The study by Elgazzar *et al*.^12^ (Egypt), was identified to be potentially fraudulent. On 15 July 2021, their study was retracted from pre-print server Research Square due to ‘ethical concerns’. It has been reported that the data for approximately 79 participants were duplicates, some deaths were recorded on dates before the trial had started and instances of plagiarism were also identified in the text.

The trial conducted by Shoumann *et al*.^13^ (Egypt) was rated as having a high risk of bias. On detailed evaluation, several methodological flaws were identified in this study. Firstly, the control arm was terminated halfway through the trial but the treatment arm was continued. This may have caused non-concurrent randomization of participants and led to a significant difference in the size of the intervention arms. This change in methodology was not reported in the trial registry. Secondly, RT–PCR tests were only performed for 12% of participants in the control arm and 2% of participants in the treatment arm, due to challenges with obtaining the required number of RT–PCR tests. For the other participants, COVID-19 was detected by checking for symptoms or using clinical tests, which are not as precise as RT–PCR tests. These variations may have resulted in significant differences between the intervention arms.

The trial conducted by Chahla *et al*.^14^ (Argentina), was rated as having a high risk of bias. Following a comprehensive assessment, some discrepancies were identified in the reported results. Some values stated in the tables differed from what was in the text of the paper. Additionally, they evaluated both healthcare and non-healthcare workers and there was a significant difference in the allocation of these participants to the two intervention arms. These variations may have resulted in significant differences between the arms.

The trial conducted by Seet *et al*.^15^ (Singapore), was rated as having some concerns of bias. This was a complex study, which involved five treatment arms. Participants in this trial were randomized using a cluster randomization method. However, results were not reported based on these clusters. According to the results presented in Table 1, hydroxychloroquine, zinc plus vitamin C, povidone-iodine and vitamin C alone were more effective than ivermectin at preventing RT–PCR-confirmed COVID-19 infection. However, it was observed that ivermectin was more effective than hydroxychloroquine, zinc plus vitamin C, povidone-iodine and vitamin C alone at preventing symptomatic COVID-19 infection. Therefore, their results for ivermectin in the prevention of COVID-19 were inconsistent.

### Meta-analysis results

**Figure 1.**
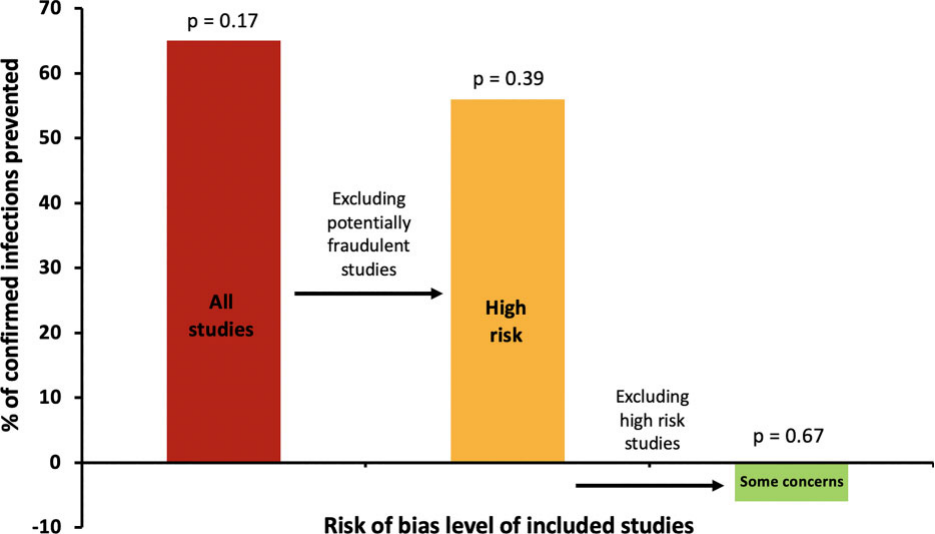
Effect of ivermectin on the prevention of confirmed COVID-19 infection.

In the meta-analysis for prevention of RT–PCR-confirmed COVID-19 infection, three studies were included (Table 1). On including all three studies, ivermectin did not have a significant effect on preventing confirmed infections (*P* = 0.17) (Figure 1 and Figure S1, available as Supplementary data at *JAC* Online). When the potentially fraudulent Elgazzar *et al*.^12^ study was excluded, ivermectin did not have a significant effect on preventing confirmed infections (*P* = 0.39) (Figure 1 and Figure S2). On excluding the high-risk Chahla *et al*.^14^ study, ivermectin failed to prevent confirmed infection in comparison with control (*P* = 0.67) (Figure 1 and Figure S3).

In the meta-analysis for prevention of symptomatic COVID-19 infection, two studies were included (Table 1). On including both studies, ivermectin did not have a significant effect on preventing symptomatic infections (*P* = 0.07). On excluding the high-risk Shoumann *et al*.^13^ study, there was one study remaining. This study, by Seet *et al*.,^15^ had multiple arms and varying endpoints. Based on this study, ivermectin had a significant effect on preventing symptomatic infections (*P* = 0.003).

## Discussion

In this meta-analysis, the use of ivermectin was not associated with the prevention of RT–PCR-confirmed COVID-19. Ivermectin had a significant effect on the prevention of symptomatic COVID-19 infection, which was based on a single study with multiple arms and inconsistent results across endpoints. Overall, three out of the four randomized trials evaluating ivermectin for the prevention of COVID-19 are at a high risk of bias or potentially fraudulent.

Non-randomized trials have also been conducted to assess the effect of ivermectin on the prevention of COVID-19. However, there are concerns about the quality of some of these studies as well. For example, an observational study by Héctor *et al*.,^16^ which suggested a 100% benefit for ivermectin in the prevention of COVID-19, has been suggested to be unreliable. Firstly, several discrepancies were identified between the registry, graphs and text in the paper for this trial. Additionally, a hospital described as being a site for this study has denied any participation. Furthermore, the raw data for this study were revealed to have duplicates for several participants and were inconsistent with results provided in the paper.

Lastly, there are real-life epidemiological surveys, where infection rates were analysed in countries including Peru and Brazil, which recommended ivermectin for use as prophylaxis.^17^ However, there are several confounding factors that make it challenging to assign cause and effect from such epidemiological surveys. For example, any reduction in COVID-19 infection rate following the recommendation of ivermectin could also be due to herd immunity, lockdown or vaccinations. We cannot use these examples as definite evidence for the efficacy of ivermectin as a preventive measure.

This suggests that the available evidence is insufficient to make a recommendation about ivermectin for the prevention of COVID-19. In order for COVID-19 vaccines to receive regulatory approval, there had to be evidence from large high-quality randomized trials that were independently audited by regulatory authorities. At this moment, we do not have such evidence for ivermectin in the prevention of COVID-19. Currently, there are multiple trials in progress, but we are not aware of any encouraging results so far.

## Supplementary Material

dkac052_Supplementary_Data

## Supplementary data

Figures S1 to S3 are available as Supplementary data at *JAC* Online.
