# Supplementary material for: Ivermectin for the prevention of COVID-19: addressing potential bias and medical fraud
Source: J Antimicrob Chemother. 2022 Feb 22;77(5):1413–6. doi: 10.1093/jac/dkac052 (PMC9326581; doi:10.1093/jac/dkac052)
Supplement: dkac052_Supplementary_Data [file dkac052_supplementary_data.docx]

**Supplementary data**


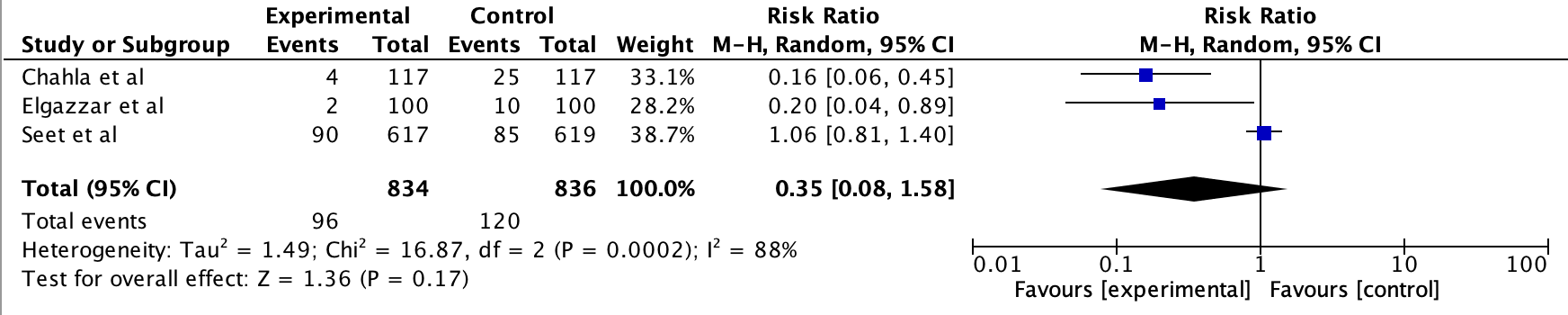

**Figure S1:** Effect of ivermectin on the prevention of RT-PCR confirmed infection including all three studies


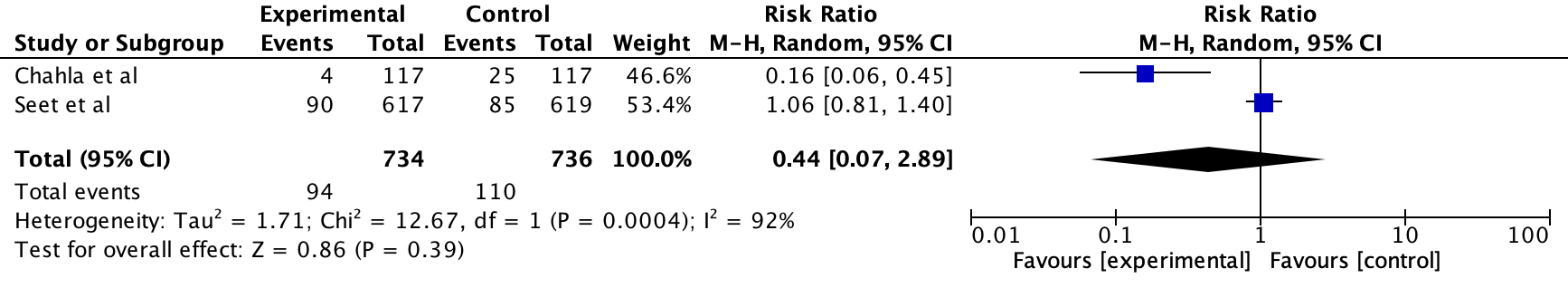

**Figure S2:** Effect of ivermectin on the prevention of RT-PCR confirmed infection excluding potentially fraudulent Elgazzar et al study


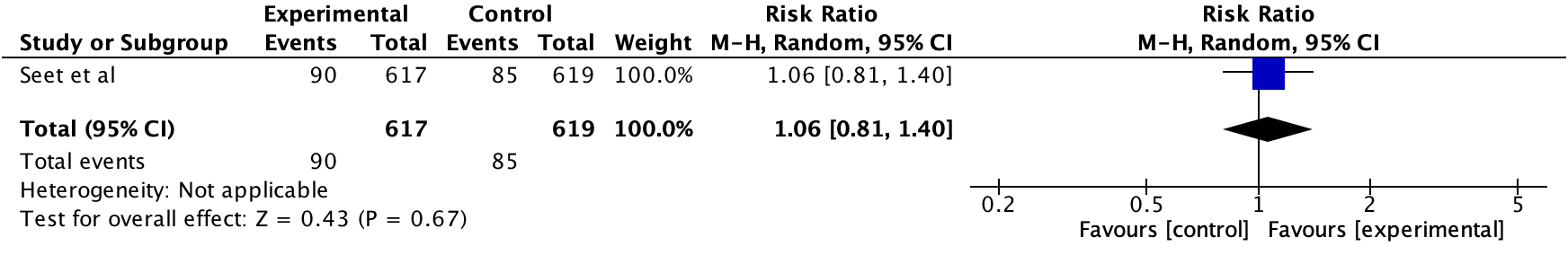

**Figure S3:** Effect of ivermectin on the prevention of RT-PCR confirmed infection excluding the high risk Chahla et al study
